# Nasal Catarrh

**Published:** 1881-04

**Authors:** B. T. Mouser

**Affiliations:** San Francisco


NASAL CATARRH.—Three Cases, with Treatment. By B. T. Mouser, M.D., San Francisco.

H. B., æt. 14, exposed in occupation to constant inhalation of fine emery. Patient called on me in April (1880), complaining of offensive breath, occasional headaches and epistaxis. The fossæ dry and a lack of secretion, epiphora caused by closure of the punctæ lachrymalis. On examination anteriorly with nasal speculum and posteriorly with small mirror, much after the style commonly used in laryngeal examinations, the fossæ are found to be roomy, pituitary membrane dry and lacking that glistening appearance seen when there is sufficient mucus. On the posterior aspect of the septum and margin of the turbinated bones a varying amount of thin, white crusts are seen, which it is impossible for patient to free himself of, by hawking down the throat or blowing the nose. Expired air is very offensive, so much so that the disagreeable odor is noticed immediately on patient's entering the room. Sense of olfaction so thoroughly blunted that this most obnoxious symptom of the disease is not apparent to himself, but is often mentioned by his friends and relatives. The punctæ lachrymalis being opened, after the lapse of three or four days all indications of epiphora disappeared; it not being necessary to dilate the canaliculi or sacs. Nasal douche was used for many months, and application of the numerous disinfectants made—carbolic acid and glycerine, permanganate of potash, salicylic acid—all proving of no avail. Pellets of cotton-wool, saturated with iodoform, carbolic acid and vaseline, were placed in the superior meatus from behind, and removed every second day. Iodoform acts on the pituitary membrane as a tonic and stimulant, and while it is a disinfectant, its unpleasant odor is disguised by the remaining two ingredients. After the removal of each pellet, the full expanse of pituitary membrane was thoroughly washed with a spray of the ten per cent. boracic acid solution. During the first month of treatment it was frequently found necessary to remove some deposits of incrustation on the turbinated bones by means of brush introduced into the posterior nares.

For some time the patient has been free from headaches and epistaxis, the offensiveness of expired air is not apparent to members of his family, and during the last two months the most thorough examinations have not revealed any fetid and tenacious crusts.

*Case* II.—R. B., æt. 50, has had considerable discharge from both nares for some time, and other disagreeable symptoms of an attack of chronic coryza; sense of olfaction augmented, and respiration through nares becoming more and more difficult; speech affected with a nasal twang; expired air not offensive. On microscopic examination, the nares were found occluded with a thick, semi-gelatinous substance; this was loosened with a spray of carbolic, boracic acid and diluted glycerine. After thorough cleansing, the right naris was found to be closed by a shining, gelatinous body, movable with a probe, and working its passage further on into the meatus upon sharp and violent expiration; the left naris being closed with the finger. Left naris posteriorily presented shining spots in middle and inferior meatuses. Pharyngitis sicca well marked. I removed two polypi, each the size of a large bean, from middle and inferior meatuses of left side, by means of wire écraseur introduced anteriorly; bleeding slight and pain moderate; base of each pedicle burnt with galvano-cautery, pellets of cotton wool saturated with olive oil, inserted and held in position by piece of dry cotton. Patient returned on second day much relieved as to difficulty of breathing, pituitary membrane of left side being considerably swollen, in consequence of the cauterization, but allowing easy respiration. The seventh day after the first operation, I removed a large polypus from inferior meatus of right side, using the écraseur as before. The écraseur was pushed through the inferior meatus from in front until it struck the wall of the pharynx, the ordinary laryngeal forceps introduced through the mouth, and the wire noose enlarged and slipped over the polypus; laryngeal forceps then removed, the pedicle crushed, and the growth removed anteriorly; base of pedicle cauterized as before. This polypus was three quarters of an inch in length and width, and of proportionate thickness, with a pedicle half an inch in length, but the latter I imagine was somewhat elongated by traction of the instrument. Cotton wool, saturated with oil, inserted as after former operations, the left naris now being open; on second day, cotton pellet removed. Slight congestion of membrane of this side, that of the left having subsided.

After a lapse of two months, patient is entirely free from trouble; pharyngitis sicca is disappearing under use of mild astringent applications, and the return of free respiration by the nares. Patient using iodide of potash daily in small doses, in order to promote a healthy action of pituitary membrane.

*Case* III. C. W., æt. 34; has suffered from chronic coryza for many years, with loss of sense of smell; headaches so severe and prolonged that life has become a burden. Examination per specula and rhinoscopic, shows general hypertrophy of mucous membrane, both nares being alternately occluded; the inferior turbinated bones being much enlarged and encroaching upon the septum, secretions are often so packed between these swellings of the membrane that forcible injections of water are made in order to give relief. Patient has used nitrate of silver in solution, lunar caustic and, in addition, every patent (proprietary) preparation in the country, but with no relief.

After repeated burnings with galvano-cautery, I found that before the inflammation following one application had subsided the remainder of the membrane on that side of the nose was much aggravated by the treatment. I then concluded to do some bold surgery, and with a strong pair of scissors cut off a considerable quantity of the middle and inferior turbinated bones, cauterizing the raw surface after the hæmorrhage had subsided. Each naris was operated on separately with an interval of ten days. Patient suffering after each operation, he was confined to his bed for forty-eight hours, and kept under the influence of opiates; the nares being alternately stuffed with oiled cotton-wool. Patient now having greater freedom of respiration, feels something of the pleasure of life, and does not think the accumulation of mucus any inconvenience. The cicatrized portion of membrane has not shown any tendency to hypertrophy since the subsidence of inflammation immediately following the cauterization.

These three cases, to my mind, are interesting as showing well-marked results and complications of acute and chronic coryza; and the perfect failure of treatment with many vaunted infallible remedies of the day. While our treatment of nasal catarrh is often unsatisfactory, it is nevertheless our duty to give our patients some careful attention, and be cautious in advising the use of quack remedies which often are not harmless.—*San Francisco Western Lancet*, January, 1881.

